# Documentation of International Classification of Headache Disorders Criteria in Patient Medical Records: A Retrospective Cohort Analysis

**DOI:** 10.7759/cureus.52209

**Published:** 2024-01-13

**Authors:** Michelle Pershing, Omkar Hirekhan, Azfar Syed, John O Elliott, Jonathan Toot

**Affiliations:** 1 Research Institute, OhioHealth, Columbus, USA; 2 Hospital Medicine, OhioHealth, Columbus, USA; 3 Hospital Medicine, CLS Health, Webster, USA; 4 Family Medicine, Soin Family Practice, Kettering Health Network, Beavercreek, USA

**Keywords:** international classification of headache disorders, outpatient clinics, headache diagnosis, headache disorders, headache

## Abstract

Objective: To determine headache diagnosis and treatment patterns in the outpatient setting, focusing on documentation of the International Classification of Headache Disorders (ICHD) criteria.

Design, setting, and participants: Retrospective cohort data were collected from electronic medical records of adults aged 18-35 who presented to resident-staffed family medicine outpatient clinics in the Midwest, USA, for a new or worsening headache between 2015 and 2016. Diagnosis codes were used to summarize the overall nature and prevalence of headaches. A random subset of 30 patients each for migraine headache (MGH) with and without aura and tension-type headache (TTH) were reviewed to determine how many of the five possible ICHD criteria were documented. Demographics/clinical characteristics, ICHD criteria, number and type of medications, and healthcare utilization (imaging, primary and emergency department care) through one year following the initial visit were summarized and compared across headache types.

Results: There were 716 unique patients during the study period (414 MGH, 227 unspecified headaches, 75 TTH, or others). Complete ICHD criteria were documented for two patients in total. There was partial documentation (e.g., one to four of the possible five) for 30% of TTH, 63% of MGH without aura, and 77% of MGH with aura (p<0.05). Across headache types, patients were prescribed an average of 2.3 to 3.3 medications over one year, with MGH patients generally trying more medications (up to eight for those with aura and up to 12 for those without). Abortive or rescue medications were prescribed to nearly all patients; prophylactics were prescribed for 50% of MGH with aura, 66.7% of MGH without aura, and 53.3%. Non-pharmacologic interventions were less prescribed: 33.3% of TTH patients and 3.3% of MGH types combined (p<0.05). Healthcare utilization was highest for MGH with aura (ED visits) and without aura (clinic visits) patients compared to TTH (p<0.001).

Conclusion: Headache-related documentation is often incomplete, which may limit interpretation and associations between diagnoses, prescribing patterns, and healthcare utilization. Future studies should evaluate the use of electronic medical records (EMR)-based templates to improve documentation, and additional detailed studies are needed in the local setting to determine whether treatment, including the use of non-pharmacologic and prophylactic methods of treatment, is optimal.

## Introduction

Headache disorders have an estimated global prevalence of 52.0%, making them among the top 15 causes of disability with disproportionate effects on women, young to middle-aged adults, and economically disadvantaged people [1, 2]. Approximately 90% of headaches are considered primary (migraine, tension-type, or cluster headaches) and have no known association with existing disease or neurologic pathology [3].

Primary headaches result in a significant burden through reduced quality of life, disability, absence from work or reduced productivity at work, and direct monetary costs from healthcare utilization, medications, and additional treatments [4]. In the United States, headache sufferers may be absent from work up to seven times more often than the national average of four days per year [5, 6], and those who go to work with a headache have reduced productivity, costing billions of dollars [7, 8]. Total treatment costs for all types of primary headaches combined are unknown but may be in the thousands to tens of thousands of dollars for patients with migraine [9]. Health-related quality of life for those with primary headache syndromes, as evidenced by changes in physical, social, and emotional well-being, is reduced to levels similar to those of patients with chronic organic diseases such as hypertension, diabetes, and congestive heart failure [5, 10].

Headache diagnosis is made clinically, often by primary care physicians (PCP), since patients are more likely to schedule with their PCP than with a neurologist or headache specialist [11]. Despite the high prevalence of primary headache syndromes in the published literature, diagnosis remains challenging, and headaches may be underestimated and misdiagnosed. There are myriad publications outlining headache diagnosis in the primary care setting [12-15], but little information exists on how often diagnoses are made using specific recommended guidelines or documenting diagnosis algorithms in the electronic medical records (EMR) [16]. In particular, it is unclear how often primary care providers are utilizing the International Classification of Headache Disorders (ICHD) guidelines published by the International Headache Society (IHS). The ICHD guidelines are considered a gold standard for headache diagnosis/classification [17-19], and following the ICHD algorithm is associated with more accurate and timely headache diagnosis [20]. While it is unclear from the literature how often ICHD algorithms are used in primary care headache diagnosis, the literature indicating that headaches are often misdiagnosed led us to hypothesize that ICHD criteria are not consistently/routinely utilized in the outpatient setting. The purpose of this study was to add to the literature regarding the documentation of ICHD criteria in patient medical records from a resident-staffed family medicine outpatient clinic. Healthcare utilization, including imaging, medications, and visits to primary care and emergency departments, was also explored.

## Materials and methods

Study setting and participant identification

This retrospective cohort study was conducted at OhioHealth, Columbus, OH, a large community-based healthcare system serving urban and suburban populations in central Ohio, USA.

The study sample was identified by a SAS-based query of institutional electronic medical records (EPIC CareConnect). The query was conducted by an informaticist independent of the study team and included patients aged 18-35 years who presented to either of two resident outpatient clinics for a new or worsening headache between March 1, 2015, and April 1, 2016. Non-diagnostic appointments (e.g., medication refill requests) left 1,170 headache-related visits for 716 unique patients in the period of interest.

Data collection

Demographic and clinical characteristics, including age, sex, race, and headache diagnosis codes, of all patients who presented with a new or worsening headache (n = 716), were pulled from the EMR.

A subset of 30 patients for each of three primary headache types: migraine headache (MGH) with aura, MGH without aura, and tension-type headache (TTH) were randomly selected for an in-depth chart review using a standardized data abstraction tool. Randomized sample selection and standardized data abstraction tools were used to minimize the potential bias associated with retrospective chart reviews. There was no a priori power analysis, as the study was exploratory in nature; the sample size of 30 charts per headache type (90 charts total) was selected based on resource availability. This selection represents approximately 40%-70% of the total study sample for each headache type and is therefore considered adequate for providing reliable point estimates.

Charts were reviewed to determine diagnosing and treatment patterns and healthcare utilization for one year following the index visit (i.e., through April 2017). Variables abstracted directly from the EMR included whether the index visit was for a new or worsening headache (patient had an existing headache diagnosis with worsening or unresolved symptoms), types of imaging ordered during the index visit (i.e., X-ray, MRI, and/or CT), whether a referral to a specialist was made, whether the patient was asked to return for follow-up and the total number of primary care and urgent care/emergency visits through the year following the index visit.

A manual review of charts was conducted based on ICHD criteria (Appendix A). The ICHD lists five specific criteria for diagnosis of MGH with or without aura and episodic TTH based on the elimination of headache as a secondary headache syndrome, frequency and duration of the headache, pain location (unilateral, bilateral), presence of visual disturbances, nausea, and vomiting [14]. Thirty charts per headache type of interest were reviewed to determine how many ICHD criteria (supplementary materials) were explicitly documented during the index encounter, meaning the first visit to the outpatient clinic during the study period.

To evaluate treatment patterns, abstracted treatment data included current patient medications, medication changes during the index visit, and medication changes during the one-year period following the index visit. Each patient's medication was presented based on the drug class and also summarized by groups (prophylactic and abortive/rescue). Rescue/abortive medications are combined because some classes of medications can be both (e.g., non-steroidal anti-inflammatory drugs (NSAIDs)).

Prophylactic: antidepressants (e.g., mirtazapine, amitriptyline, nortriptyline, sertraline), beta-blockers (e.g., metoprolol, propranolol), calcium channel blockers, neuronal stabilizing agents (e.g., divalproex, gabapentin, topiramate), supplements (e.g., magnesium, butterbur), muscle relaxers, trigger point injections.

Abortive/rescue: acetaminophen/aspirin/caffeine (AAC), acetaminophen, antihistamine and/or antiemetic (e.g., diphenhydramine, promethazine), butalbital/acetaminophen/caffeine (BAC), benzodiazepines, NSAIDs (including over-the-counter or prescription), opioids, steroids, and triptans.

In order to assess medication use, the number of medication types each patient was prescribed throughout the study period was summed. For example, if a person who was taking NSAIDs prior to their index visit had a triptan added at the index visit (without being instructed to stop taking NSAIDs), then a prophylactic added during the one-year follow-up would have a count of three medications for the entire study period. A person who tried three different types of triptans during the study period would also have a count of three medications. The total number of non-pharmacologic interventions prescribed during the index visit or the one-year follow-up was also recorded.

Data analysis

Data were described using frequencies and percentages for categorical variables and means and standard deviations, or medians and ranges, for continuous variables. Comparisons of continuous data between groups of interest were made using a one-way ANOVA followed by Tukey’s honestly significant difference (HSD) test for normally distributed data or the Kruskal-Wallis test followed by the Mann-Whitney U-test for non-normally distributed data. Categorical data were compared using the Chi-square or Fisher’s exact test if any cell sizes were less than five. P-values <0.05 were considered statistically significant.

## Results

Overall headache-related visits

Over the 13-month study period, 716 unique patients presented to an outpatient clinic for a new or worsening headache. Demographic characteristics of the patients are shown in Table 1.

**Table 1:** Demographic characteristics of patients with documented headache-related visit

Demographic characteristics, n (%)	n=716
Age, mean (SD)	28 (5.0)
18-23 years, n (%)	160 (22.3)
24-29 years, n (%)	261 (36.5)
30-35 years, n (%)	295 (41.2)
Female, n (%)	590 (82.4)
Race, n (%)	
Caucasian	532 (74.3)
Black/African American	116 (16.2)
Other/declined	68 (9.5)
Headache diagnosis, n (%)	
Migraine	414 (57.8)
Unspecified migraine	290 (70.0)
Migraine without aura	79 (19.1)
Migraine with aura	42 (10.1)
Other migraine types	3 (0.7)
Headache not otherwise specified	227 (31.7)
Tension	67 (9.4)
All others	8 (1.1)

The majority of patients were Caucasian (74.3%), female (82.4%), and 24-35 years of age (77.7%). Based on International Classification of Diseases, Tenth Revision (ICD-10) diagnosis codes, the most common diagnosis in the outpatient setting was unspecified migraine (40.5%) followed by headache (31.7%), MGH without aura (11.0%), TTH (9.4%), MGH with aura (5.9%), and all others (other migraine types, new daily persistent headache, cluster headache; 1.5% combined).

Subset analyses

As shown in Table 2, the demographic characteristics of the subset of patients (30/headache type) with MGH with aura, MGH without aura, and TTH selected for in-depth chart review were consistent with the system-wide population, with the majority of patients being female (83.3%) and Caucasian (63.3%).

**Table 2:** Demographics and characteristics of a subset of headache patients ^a^ Significantly more visits were for worsening symptoms than a new headache, p<0.001; w/: with; w/o: without

	Total N=90	Migraine w/ aura N=30	Migraine w/o aura N=30	Tension N=30
Age, mean (SD)	27.9 (4.4)	27.0 (4.82)	27.9 (3.64)	29 (4.62)
Female, n (%)	75 (83.3)	24 (80.0)	28 (93.3)	23 (76.7)
Body mass index, mean (SD)	30 (8.1)	28 (7.3)	32 (8.8)	30 (8.2)
Reason for the visit, n (%)				
Worsening symptoms	66 (73.3)^a^	24 (80.0)	30 (100.0)	12 (40.0)
New headache	24 (26.7)	6 (20.0)	-	18 (60.0)
Physician				
Attending	65 (72.2)	23 (76.7)	17 (56.7)	25 (83.3)
Resident	25 (27.8)	7 (23.3)	13 (43.3)	5 (16.7)

The majority of index visits for migraine (80% of MGH with aura visits and 100% of MGH without aura visits) were for worsening/unresolved headaches. Index visits for patients with TTH were split between new headache visits (60%) and worsening/unresolved headaches (40%).

International Classification of Headache Disorders documentation

**Figure 1:**
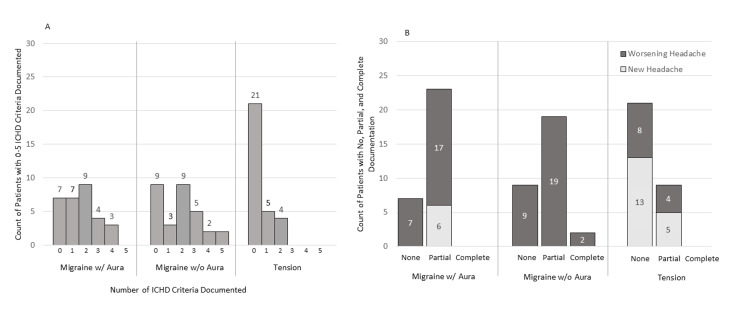
Summary of the ICHD criteria Panel A shows how many ICHD criteria were documented for each headache type. Panel B collapses ICHD criteria into none, partial, and complete, by headache type and visit type. Overall, there was a significant difference (p<0.001) in ICHD documentation based on headache type. Documentation for tension-type headaches was significantly lower than for either type of migraine (p<0.05). There was no statistical difference in the level of documentation by visit type for new versus worsening headaches (p = 0.287). ICHD: International Classification of Headache Disorders; w/: with; w/o: without

There was a significant difference (p<0.001) in ICHD documentation based on headache diagnosis, with no, partial, and complete documentation occurring for 23.0%, 77%, and 0% of MGH with aura, 30%, 63%, and 7% of MGH without aura, and 70%, 30%, and 0% of TTH (Figure 1).

Documentation for TTH was significantly lower than for either MGH type (p<0.05). There was no difference in the level of documentation based on whether the visit was for a new versus worsening headache (p=0.287).

Treatment patterns

In order to assess medication use, the number of medication types each patient used throughout the study period was summed, and a one-way ANOVA was performed to compare the total number of medications across the three headache groups of interest. There was no significant (F2, 87=2.03; p=0.137) difference in the mean number of medications per person through one year after diagnosis between patients diagnosed with MGH with aura (3.3 ± 2.03; 95% CI (2.54, 4.06)), MGH without aura (3.0 ± 2.25; 95% CI (2.16, 3.84)), or TTH (2.3 ± 1.34; 95% CI (1.80, 2.80)). There was a significant difference in the number of non-pharmacologic interventions, with 33.3% of TTH patients prescribed or recommended non-pharmacologic options compared to 3.3% of MGH types (one patient each in the MGH with and without aura groups; p<0.05).

Of the 90 patients evaluated, all but one (TTH) were prescribed at least one abortive or rescue medication at some point between the index visit and the first year of follow-up. The percentage of patients on any type of prophylactic was 15/30 (50%) for MGH with aura, 20/30 (66.7%) for MGH without aura, and 16/30 (53.3%) for TTH.

Treatment patterns among headache types of interest are outlined below.

Migraine With Aura

**Figure 2:**
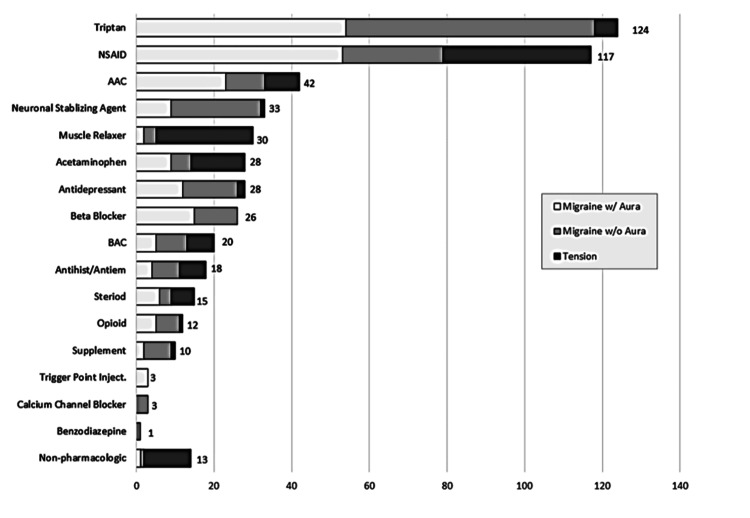
Summary of medication types used across all headache types and visits The type of medication each patient was prescribed from the pre-index visit through one year of follow-up was counted. Triptans and NSAIDs were the medication types most commonly used. Other commonly prescribed abortive medications included acetaminophen or acetaminophen-combination medications. Steroids and opioids were prescribed less frequently. Commonly used prophylactic medications included antiepileptic/anticonvulsant medications, muscle relaxers, beta-blockers, and various antidepressants. Supplements (e.g., magnesium), trigger point injections, calcium channel blockers, and benzodiazepines were rarely prescribed. NSAID: non-steroidal anti-inflammatory drugs; AAC: acetaminophen/aspirin/caffeine; BAC: butalbital/acetaminophen/caffeine; inject.: injection

The total number of medications among MGH with aura patients ranged from one to eight medications over the entire study period, i.e., from index through one year of follow-up: 66.7% (n=20) tried one to three medications; 26.7% (n=8) tried four to six medications; and 6.7% (n=2) tried >6 medications. The most common treatment regimens/medication types for MGH with aura were abortive/rescues (triptans), NSAIDs, AAC, and preventatives like neuronal stabilizing agents, antidepressants, and beta-blockers (Figure 2).

During the index visit, medication changes were made for the majority of patients (25/30; 83.3%). Of those with medication changes, 50% were prescribed a new or different triptan, and 23.3% were prescribed a prophylactic. Additional rescue/abortive medications were added for 10% of MGH with aura patients.

Follow-up visits in the one-year period after the index visit occurred for 18/30 (60.0%) patients with aura. Of these, nine (50%) had additional medication changes during follow-up, comprising adding or changing triptans and adding prophylactic medications. Only one patient was prescribed a non-pharmacologic option (massage).

Migraine Without Aura

The total number of medications among MGH without aura patients ranged from one to 12 medications: 80.0% (n=24) tried one to three medications; 13.3% (n=4) tried four to six medications; and 6.7% (n=2) tried >6 medications over the entire study period, i.e., from index visit through one year of follow-up.

All patients diagnosed with MGH without an aura presented with a worsening headache. Patients were commonly (25/30; 83.3%) already taking abortive/rescue medications. Use of prophylactics was noted for 9/30 (30%) of those without aura. There were no medication changes suggested following medication reconciliation at their visit for 17/30 (56.7%) of patients diagnosed with MGH without aura. Of those with medication changes, the majority (9/13; 69.2%) were prescribed a new or different prophylactic, and six were prescribed a new or different abortive and/or rescue medication.

Follow-up visits in the one-year period after the index visit occurred for 27/30 (90.0%) migraine without aura patients. Additional medication changes were noted for 16/27 (59.3%) patients during follow-up: seven discontinued all medications, seven had medication additions or changes within the class, and two patients had multiple changes over several visits. Only one patient was prescribed a non-pharmacologic option (cognitive behavioral therapy).

Tension-Type Headache

The total number of medications among TTH patients ranged from 0 to seven medications: 3.3% (n=1) tried no medications; 80.0% (n=24) tried one to three medications; and 16.7% (n=5) tried four or more medications.

Among those with TTH, 18 (60.0%) presented with a new headache, and 12 (40.0%) presented with a worsening headache. The majority of those presenting for a new headache were not on any medications prior to the index visit, and all were prescribed one or more medications, including muscle relaxers (9/18), NSAIDs (9/18), neuronal stabilizing agents (1/18), triptans (1/18), supplements (1/18), steroids (1/18), or antiemetics (1/18). Patients presenting with worsening headaches were commonly already taking analgesics (11/12); triptan and muscle relaxer use were limited to one patient each. All but one patient who presented with a worsening headache were prescribed new medications, including adding or changing analgesics (4/12), adding steroids (3/12), adding antihistamines (1/12), adding or changing muscle relaxers (3/12), and adding tricyclic antidepressants (TCA) (1/12).

Follow-up visits in the one-year period after the index visit occurred for 13/30 (43.3%) patients diagnosed with TTH. Additional medication changes occurred for six of them: add or change muscle relaxers (3/6), add or change analgesics (4/6), add antidepressants (1/6), add triptans (1/6), add opioids (1/6), and add antiemetics (1/6). Non-pharmacologic options were prescribed for 11/30 tension headache patients (e.g., massage/physical therapy, chiropractor, home range of motion or stretching, and/or osteopathic manipulative therapy).

Healthcare utilization

Specialist Referral and Imaging Studies

Slight, non-statistically significant differences were noted in referral patterns based on the type of headache diagnosis and type of visit. Only 10% of patients diagnosed with TTH (95% CI (0.04, 0.26)) were referred to specialists when compared to 23% of patients diagnosed with MGH with aura (95% CI (0.12, 0.41)) and 27% of MGH without aura patients (95% CI (0.14, 0.45); p=0.233). Patients were more likely to be referred to a specialist, such as a neurologist or pain management specialist if they had a worsening headache versus a new headache (37.1% versus 13.5%, respectively).

**Figure 3:**
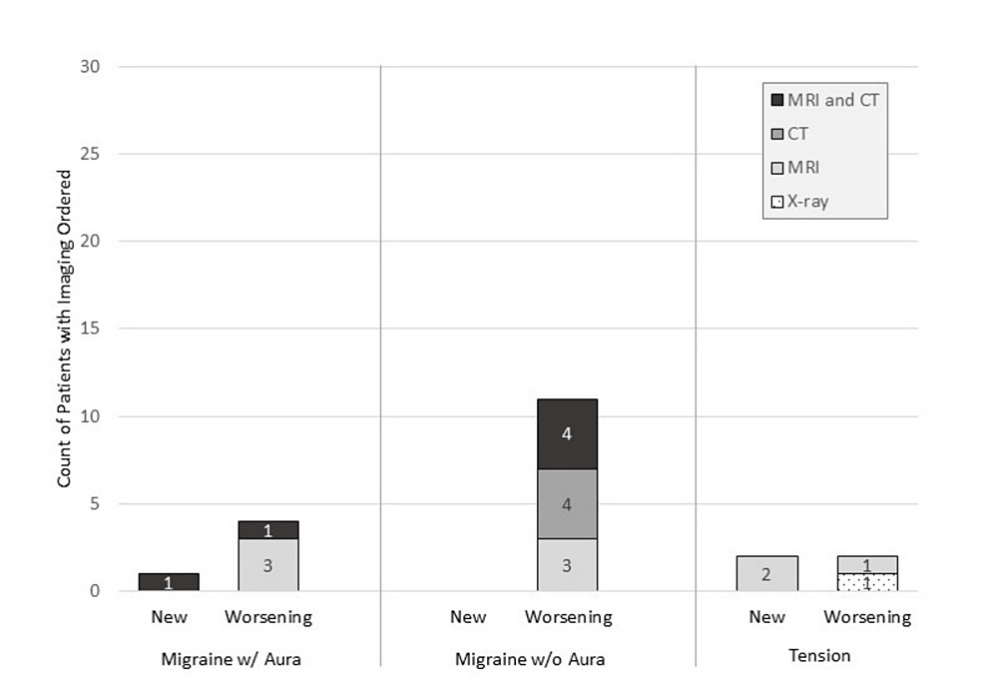
Summary of imaging ordered at the index visit Imaging was ordered for 20 of 90 patients who presented to an outpatient clinic for a headache. Imaging was ordered more often for worsening headaches than new headaches and migraines without aura than for other headache types; however, the differences were not statistically significant. w/: with; w/o: without

Imaging was ordered for 5/30 (16.7%), 11/30 (36.7%), and 4/30 (13.3%) of patients diagnosed with MGH with aura, MGH without aura, and TTH, respectively (Figure 3).

Imaging was ordered for 25.8% of those with worsening symptoms and 12.5% of those with new headaches. Of the 20 patients with imaging ordered, 45% were for MRI alone, 30% were for MRI plus CT, 20% were for CT alone, and 5% were for X-ray. The majority of patients (60.0%) who had imaging ordered were also referred to a specialist.

Primary Care and Emergency Department Utilization

**Figure 4:**
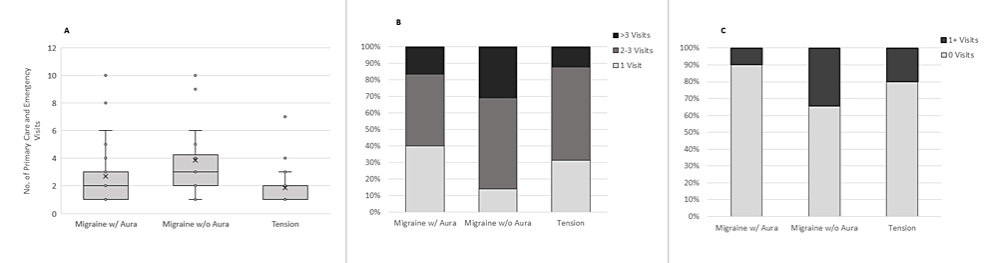
Healthcare utilization Panel A: There was a statistically significant (p<0.001) difference in overall healthcare utilization (e.g., PCP plus ED visits) based on headache type. Patients with TTH had significantly fewer visits (1.9 ± 1.4) than MGH with aura (2.7 ± 2.2) and MGH without aura (3.8 ± 2.7); p<0.001. Panel B: Healthcare utilization was driven primarily by primary care visits. Patients with MGH without aura had the highest primary care utilization, with 30% of patients having more than three primary care visits in one year, compared to 16.7% of MGH with aura patients and 6.7% of TTH patients (p<0.05). Panel C: Patients with MGH with aura had higher emergency department utilization as well, with 34.4% of patients visiting the ED at least once, compared to 10% of MGH with aura and 20% of TTH, although the difference was not statistically significant (p=0.076). PCP: primary care physicians; TTH: tension-type headache; MGH: migraine headache; w/: with; w/o: without

There was a statistically significant (F2,87=25.533; p<0.001) difference in overall healthcare utilization (e.g., PCP plus ED visits) based on headache type (Figure 4).

The TTH patients had significantly fewer visits (1.9 ± 1.4; 95% CI (1.38, 2.42)) than MGH with aura (2.7 ± 2.2; 95% CI (1.88, 3.52)) and MGH without aura (3.8 ± 2.7; 95% CI (2.79, 4.81)); p<0.001.

The total number of PCP visits over one year for patients in the MGH with aura group, MGH without aura group, and TTH group was 77, 98, and 50, respectively. The MGH without aura patients had significantly higher primary care utilization than patients diagnosed with MGH with aura or TTH (p<0.05). Thirty percent of the MGH without aura patients had more than three primary care visits, compared to 16.7% of the MGH with aura and 6.7% of TTH patients (Figure 4).

The total number of emergency room visits for patients in the MGH with aura group, MGH without aura group, and TTH group was 4, 17, and 6, respectively. The MGH without aura patients and TTH patients were slightly more likely to use the emergency room than the MGH with aura patients: 33.3%, 20.0% for the MGH without aura and TTH, and 10% of MGH with aura patients visited the emergency room at least once (p=0.076) (Figure 4).

A request for follow-up was documented for 6/30 (20.0%) of MGH with aura, 29/30 (96.7%) of MGH without aura, and 17/30 (56.7%) of TTH patients (p<0.001). The MGH without aura patients were significantly more likely to be asked to follow up than those diagnosed with MGH with aura or TTH. In alignment with PCP requests to follow up, there was a significant difference in whether or not patients actually did follow up: at least one follow-up visit was completed for 18/30 (60%) of MGH with aura, 26/30 (86.7%) of MGH without aura, and 11/30 (36.7%) of TTH patients (p<0.001).

## Discussion

Headaches are very common and can be difficult to diagnose and effectively manage, often relying on subjective information provided by the patient. The use of a structured algorithm such as the ICHD criteria would likely improve diagnosis. It is unclear how often this type of structured algorithm is used or documented in an outpatient setting. Therefore, the overall purpose of this study was to evaluate headache documentation, treatment, and healthcare utilization for headache-related visits conducted at resident outpatient clinics. The results of this study highlight several important points regarding headache patient demographics, diagnostic documentation, abortive vs. prophylactic headache treatment, and potentially increased healthcare utilization.

The general demographic findings, when compared to larger studies, are consistent with Caucasian females of childbearing age having more headaches overall, particularly migraines. A priori, our focus for in-depth chart review for this study was migraine (with and without aura) and tension because they have clearly defined ICHD criteria. However, our resulting data pull showed that a majority of headaches (72.2%) were classified as unspecified migraine (ICD code G43.9) or headache not otherwise specified (ICD code R51), which do not have specific ICHD diagnosis criteria.

Although the ICHD is a widely accepted diagnosis tool for headache classification with a specificity of 96% for MGH with aura [20], there are other methods for headache diagnosis and management [15,21,22]. In our chart review, there was no clear evidence in the history of the present illness, review of systems, or assessment and plan to indicate that alternative diagnostic tools, scoring systems, or algorithms were used. As such, it is possible that the diagnoses for the patients in our cohort may be inaccurate, which may have contributed to treatment delays or failures. However, the nature of our study precludes making formal assumptions. The lack of documented criteria does not necessarily mean that differential diagnoses or red flag symptoms were overlooked or that scoring tools and other methods were not used to reach a diagnosis; the supporting documentation simply wasn’t recorded, consistent with other limited investigations of primary care EMR documentation [23].

Overall, there was minimal ICHD documentation for patients in our cohort, with 0 to two ICHD criteria out of a possible five documented for the majority of patients. Since our study included a new or worsening headache at the index visit, one possible explanation for the lack of documentation is preexisting PCP familiarity with the patient’s medical history. Alternatively, a previously made headache diagnosis may have also been carried forward with or without re-evaluation at any subsequent clinic visits; however, we found no statistical difference in the level of documentation based on a new or worsening headache.

Interestingly, while ICHD documentation was sparse, there was documentation of symptoms potentially contradictory to the diagnosis entered for 5/30 MGH with aura patients and 20/30 TTH patients. Among MGH with aura patients, there was documentation of daily frequency and/or episodes lasting greater than 72 hours, which is suggestive of a chronic migraine or other headache type [19,21]. Of note, with the exception of seven patients with new daily persistent headaches, there were no ICD codes related to chronic headaches of any type in our overall review of the two outpatient clinics. Among TTH, there was documentation of unilateral location, vision changes, nausea/vomiting, pulsing quality, severe intensity, or photo/phonophobia, which could also be suggestive of MGH.

However, are these noted differences in documentation and possibly conflicting symptomatology actually clinically relevant? A closer evaluation of the first-line pharmacologic treatments for our cohort shows some expected similarities with the literature and evidence-based best practices regarding both abortive and prophylactic medications. For example, NSAIDs, acetaminophen, and AAC are often used for headache relief in all three of the headache types evaluated, and triptans were common for the MGH subtypes. Butalbital-based medications, antihistamines and/or antiemetics, steroids, and opioids were used less often. In addition, only 25% of MGH (20.0% MGH with aura and 30.0% MGH without aura) in our study reported initially being on a prophylactic medication at the time of their index visit, which is similar to other literature reports indicating limited use of prophylactic medications [24-26]. Use of prophylactic medications increased to 50% following the index visit and 58.3% through follow-up, which suggests that the appropriate MGH treatment options for abortive and prophylactic medications were being evaluated and worked through in a stepwise fashion, even though the documentation for a corresponding change from acute to chronic MGH or first-line treatment failure was not documented.

Patients in our cohort tried one to 12 medications for MGH with aura, one to eight medications for MGH without aura, and 0 to seven medications for TTH over a one-year period. While some patients tried up to 12 medications, this was primarily sequential rather than concurrent. The majority of patients, regardless of headache type, were taking one to two medications at a time. Up to 20% of patients were taking three medications at a time, and only MGH patients took >3 medications at a time (up to seven concurrent medications). Previously published reports indicate that polypharmacy is common in almost 60% of chronic headache patients, with NSAIDs accounting for 73.5% and triptans for 49.1% [27]. Although combination medications such as triptan plus naproxen or including abortives with prophylactics can have increased effectiveness compared to monotherapy [28], increasing the number of medications used to treat headaches without finding an appropriate regimen does not necessarily improve health outcomes as it can lead to medication overuse headaches (MOH) [29].

In the context of this study, it is possible that headaches diagnosed as TTH or MGH may actually be MOH, as documented instructions to stop taking medications associated with MOH were rare. With any pharmacological management, the selection of an appropriate treatment option is based on having the correct diagnosis. Potential misdiagnosis may be perceived by the patient as treatment failure, resulting in poor patient satisfaction, transfer of care, or increased emergency department or urgent care utilization. 

The majority of treatment plans for our cohort were pharmacologic in nature. Nonpharmacologic management was documented for only 11 TTH patients and two MGH patients. Trigger identification/avoidance was also rarely documented (one MGH with aura, three MGH without aura patients) and included menstrual, weather/barometric pressure, and food triggers. There was no documentation in any records that headache diaries or other tracking/trigger identification methods were discussed.

Imaging studies, the number of clinic or ED visits, recommended follow-up clinic visits and even referrals are all different aspects involving measurements of healthcare usage. Imaging studies were ordered for 20/90 of the patients in our cohort. There was no difference in imaging ordered based on headache type, but imaging was ordered more frequently for those with worsening headaches (n=17) than those with new headaches (n=3).

We did observe emergency resource utilization, particularly among MGH, which is consistent with literature reports suggesting that headache is among the top five complaints for patients seeking emergency-level care, resulting in substantial numbers of emergency visits and hospitalizations each year in the United States [30]. One method to help reduce emergency room visits is to encourage close follow-up at the clinic with realistic expectations of headache management, for example, if starting a prophylactic treatment, which can take two months of up-titration for efficacy to be noticeable. Follow-up requests were documented for 20% of MGH with aura, 96.7% of MGH without aura, and 56.7% of TTH. The reason why MGH without aura patients were requested to follow up more often than those with aura is unclear and may simply be a spurious finding. Regardless of the reason, MGH and TTH patients who followed up in outpatient clinics were also overwhelmingly responsible for the ER visits.

There were several limitations to this study, most notably that a convenience sample of two specific resident outpatient clinics was used. Therefore, these results may not be generalizable to other settings. As noted previously, unspecified headaches/unspecified migraines comprised the majority (72.2%) of overall formal diagnoses in the outpatient setting. Because these do not have clear ICHD criteria, we focused on MGH with/without aura and TTH. However, these diagnoses comprised a smaller percentage of the overall headache burden, so we cannot say the findings are entirely representative of headache diagnosis and treatment in the outpatient setting. Although many abortive medications across the headache types evaluated in the study are similar, the overall approximately one-year study timeframe limited our evaluation of how ICHD criteria documentation, diagnosis, and treatment options fully influenced healthcare utilization. This is particularly relevant since we know that headache patients often have a delayed time to correct their diagnosis (four to seven years), and the use of IHS criteria can result in an earlier time of diagnosis [25, 26]. Healthcare utilization data only includes PCP and ED visits made within one network and may underrepresent overall usage if patients had out-of-network PCP, ED, or urgent care visits. We limited our review to patients aged 18-35 to reduce potential confounds, but this exclusion may further underestimate the overall burden of headaches in primary care. Due to the retrospective nature of the study, we were unable to determine whether patients stopped coming because headaches were resolved, because they transferred care elsewhere, or because they decided to endure the symptoms.

Of the several possible future directions for this study, there should be a focus on documentation, training, medication use, and patient follow-up. The electronic medical record EPIC has several tools to help increase effective documentation in a time-efficient manner, including the use of dot phrases, the incorporation of standardized diagnostic algorithms, and a standalone headache visit template. Further exploration of the widespread use of unspecified headache/migraine diagnosis codes would also be of interest. Additional training regarding headache diagnoses is an option, but without having more than a few examples of a potential misdiagnosis, the physician might be better suited to focus on documentation instead. There should be a focus on how to more efficiently capture the medical decision-making taking place during the clinic encounter, how to set expectations, and how to encourage follow-up, including when to refer to a specialist.

## Conclusions

There were incomplete ICHD criteria or contradictory documentation to support the diagnosis for most patients evaluated for a new or worsening headache. The majority of headache diagnosis codes in our primary care setting were for unspecified headaches or migraine, clinical/billing diagnoses that are not supported by ICHD. This may suggest that PCPs are not sufficiently utilizing ICHD criteria. Taken together, we may question the accuracy of headache diagnoses in the primary care setting. Given the number of medications used, the possibility of medication overuse headaches is a concern.

Headache disorders are one of the top 10 reasons patients schedule visits with PCPs, making primary care an ideal setting to improve diagnosis and reduce health burdens. Incorporating electronic health record builds and controls and improving residency training on headache diagnosis and treatment could result in faster, more accurate headache diagnoses. In turn, this may reduce overall healthcare utilization or at least shift utilization from emergency care to primary care.

## Appendices

Appendix A 

**Table 3:** ICHD Criteria

Migraine with Aura
1. At least two episodes fulfilling the following criteria: 2. Aura consisting of at least one of the following, but no motor weakness: Fully reversible visual symptoms including positive features (e.g., flickering lights, spots or lines) and/or negative features (i.e., loss of vision) Fully reversible sensory symptoms including positive features (i.e., pins and needles) and/or negative features (i.e., numbness) Fully reversible dysphasic speech disturbance 3. At least two of the following: Homonymous visual symptoms and/or unilateral symptoms At least one aura symptom develops gradually over five or more minutes and/or different aura symptoms occur in succession over five or more minutes Each symptom lasts at least five minutes, but no longer than 60 minutes 4. A headache that fulfills the criteria for migraine without aura, and begins during the aura or follows the aura within 60 minutes. 5. Headache is not attributed to another disorder
Migraine without aura
1. At least five episodes 2. Episodes lasting four to 72 hours (untreated or unsuccessfully treated) 3. Headache has at least two of the following characteristics (circle all that apply): Unilateral location Pulsating quality Moderate or Severe pain intensity Aggravated by (or causes avoidance of) routine physical activity 4. During the headache, the patient experiences at least one of the following: Nausea or vomiting Photophobia Phonophobia 5. Headache is not attributed to another disorder
Episodic tension-type headache
1. Headache lasts 30 minutes to seven days 2. Headache has at least two of the following features Bilateral location Pressing or tightening (non-pulsating) quality Mild or moderate intensity Not aggravated by routine physical activity such as walking or climbing stairs 3. Both of the following: No nausea or vomiting No more than one of photophobia or phonophobia 4. At least 10 episodes occurring fewer than one day per month on average (fewer than 12 days per year) or at least 10 episodes occurring on more than one but fewer than 14 days per month for at least 3 months 5. Not attributed to another disorder
